# Developing a Memory and Communication App for Persons Living With Dementia: An 8-Step Process

**DOI:** 10.2196/44007

**Published:** 2023-03-15

**Authors:** Ellen L Brown, Nicole Ruggiano, Sai Chaithra Allala, Peter J Clarke, Debra Davis, Lisa Roberts, C Victoria Framil, Maríateresa Teri Hernandez Muñoz, Monica Strauss Hough, Michelle S Bourgeois

**Affiliations:** 1 Nicole Wertheim College of Nursing and Health Sciences Florida International University Miami, FL United States; 2 School of Social Work The University of Alabama Tuscaloosa, AL United States; 3 Knight Foundation School of Computing and Information Sciences Florida International University Miami, FL United States; 4 University of South Florida Tampa, FL United States

**Keywords:** health technology, interdisiplinary team, older adults, dementia, communication, communication aids for disabled persons, communication boards, app, Alzheimer disease, family, caregiver, clinical care, development, speech, psychosocial intervention, software design

## Abstract

According to the 2022 Alzheimer’s Association Facts and Figures, more than 6 million Americans have Alzheimer disease and related dementias. They are cared for by millions of family members, friends, or other unpaid caregivers. Communication deficits are common among persons with Alzheimer disease and related dementias and pose challenges to caregiving and clinical care, which is already complex. An interdisciplinary team developed a mobile app prototype to improve communications between people living with dementia and their caregivers and providers and to promote person-centered care. This viewpoint paper provides a road map for how the interdisciplinary team worked together to develop and plan for the implementation and evaluation of a new evidence-based app. In our paper, we provide an 8-step process used by a team of clinicians, researchers, and software engineers to develop a new app to meet the needs of people living with dementia and their caregiver(s). The planned clinical trial has been registered at ClinicalTrials.gov (NCT04571502; https://clinicaltrials.gov/ct2/show/NCT04571502).

**International Registered Report Identifier (IRRID):**

RR2-10.3928/19404921-20210825-02

## Introduction

### Background

People living with dementia often experience communication deficits due to a variety of symptoms associated with Alzheimer disease and related dementias, including memory loss, primary progressive aphasia, decreased attention span, and word-retrieval anomia [1-6]. These deficits can have a negative impact on caregiving and clinical care for people living with dementia while also resulting in negative physical and mental health outcomes for their caregivers [6-8].

Prior research has found that augmentative and alternative communication (AAC) devices can effectively support people living with dementia in communicating [9]. However, many of these AAC devices are limited in their ability to customize content and have been criticized for their limitations in addressing the personhood of people living with dementia and targeting the family as a unit in communication support [9,10]. Specifically, most of these technology-based devices are not designed to support people living with dementia in communicating their daily and changing preferences and needs [9-19]. This paper describes the first phase of a clinical trial, where a team of interdisciplinary researchers applied a multistep framework for user interface (UI) development [20] to develop an electronic AAC device, the My Person Assisted Touchscreen Interface (*My PATI*) for people living with dementia.

### The My PATI for People Living With Dementia

The focus of this overall project was to develop an electronic AAC device for people living with dementia that aims to (1) support them in communicating their daily care preferences and needs with caregivers; (2) communicate their everyday experiences and behaviors with caregivers; (3) share information about these experiences and behaviors with providers involved with their care; and (4) provide the caregivers with the ability to easily update information, with internet access.

**Figure 1:**
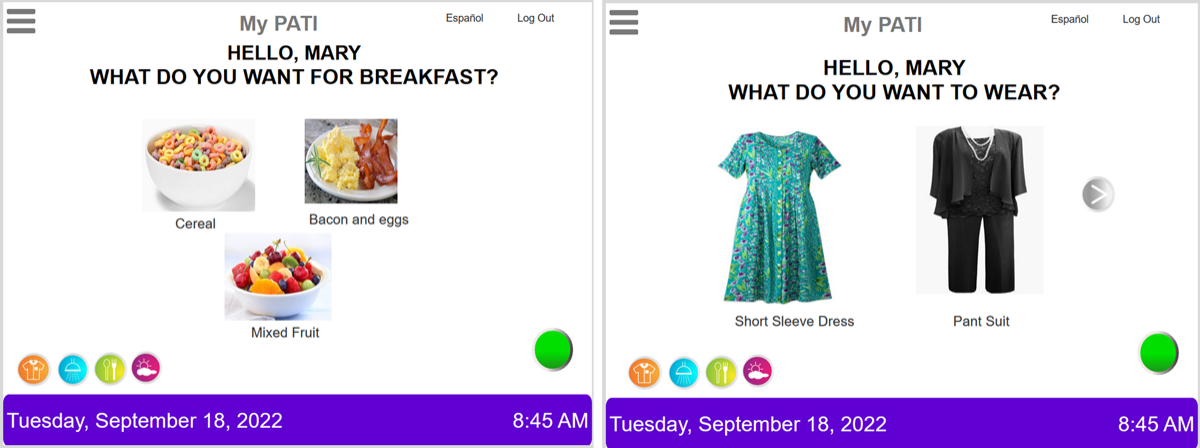
Screenshots of Mary’s preferences for breakfast and clothes. My PATI: My Person Assisted Touchscreen Interface.

The newly developed *My PATI* provides structure and prompts and serves as a tool for ongoing engagement between people living with dementia and their caregivers. Similar to traditional paper AAC devices, *My PATI* uses graphic images and text that people living with dementia can point to when trying to communicate. Unlike traditional AAC devices, these graphic images can be customized. For example, rather than a generic icon of a shirt or dress, *My PATI* can be populated with images of the care recipient’s actual clothing to make *My PATI* more relevant to the person living with dementia when communicating about getting dressed for the day (see an example in Figure 1). It also differs from traditional AAC devices in that its features are organized by activities of daily living (ADL; ie, basic self-care tasks, such as eating and grooming) and instrumental ADL (IADL; ie, more complex activities, such as preparing meals and maintaining schedules). *My PATI* is designed to support the autonomy and life participation of people living with dementia, by allowing them to make selections about their care preferences during ADL and IADL throughout the day. For example, *My PATI* provides personalized touchscreen selection options for grooming and dressing (ie, ADL) and prompts the user to communicate, manage, and engage in their surroundings (ie, IADL). The people living with dementia can use *My PATI* alone (when able to) or in partnership with a family caregiver, another family member, or a paid caregiver involved with their care.

*My PATI* has features that allow the caregiver to continually update content as the care recipient’s needs and preferences evolve, which is not easily done with a paper version of an AAC device. The caregiver UI of *My PATI* is designed to allow the caregiver to upload, add, or change graphics and functions that reflect the care recipient’s unique preferences and memorabilia. For example, images of clothing may be updated to reflect a change in the season, or a change of diet may necessitate the need for different food choices. This feature of updating graphic images and the ability to access the app from anywhere with internet connectivity is novel. Textbox 1 features the fictitious case of Mary, an older adult with dementia, demonstrating an example of the potential use of *My PATI*. Figure 1 provides a screenshot from *MY PATI,* displaying options Mary is offered regarding her breakfast preferences and what she would like to wear.

**Example use case of Mary.**

Mary is 80 years old, has multiple medical problems, and was diagnosed with vascular dementia 6 years ago. Mary is often confused and anxious and does not sleep well. Her family, including her live-in son, makes every effort to let the daily paid nursing assistants know Mary’s care preferences (eg, types of food, clothing, and grooming), interests (eg, preferred TV shows, music, daily walks, and manicures), and abilities (eg, able to select her clothing and style her hair). Mary has significant verbal communication deficits. Therefore, she often communicates through single words or short phrases, which frequently results in family members and in-home caregivers not giving Mary a choice on what to wear, eat, or do for her daily activities, despite their good intentions for her daily care. Sometimes Mary exhibits care-resistant behaviors (eg, aggression and lack of cooperation) when she seems unhappy with food or clothing suggestions. Using *My PATI*, Mary’s caregivers are able to communicate with Mary about what she wants to eat or wear by using pictures, and Mary can select them using the touchscreen rather than having to verbally communicate about them. This reduces the frequency of Mary’s behavioral disturbances, and Mary is more frequently alert and engaged during mealtimes and dressing. Essentially, *My PATI* is a tool that compensates for Mary’s communication deficits and provides a method for her to make choices that facilitates involvement in her care.

*My PATI* is grounded in evidence from low-technology versions of AAC devices [21]. In the mid-1980s, when dementia was a new diagnosis, there were no known interventions for its accompanying memory and communication problems. Bourgeois [22] developed the *Memory Wallet*, a collection of 30 pages of personal information, one photograph per page captioned with a 5- to 7-word declarative sentence, to provide prompts for desired conversation topics. Persons with midstage dementia used the wallets to read the sentence and comment about the photograph, increasing the overall number of on-topic statements made during conversations with the wallet compared to those without the wallet; they also decreased the number of ambiguous, repetitive, and erroneous utterances made during conversations with the wallet. In the following years, Bourgeois [23] systematically replicated the effects of the 1990 study with individuals across the continuum of dementia severity using various sizes of memory books and demonstrated similarly positive effects, as long as the size of the font was large enough to be read easily, and the content of the pages was personally relevant. Caregivers reported serendipitous effects of the memory aids on other challenging behaviors, including the reduction of repetitive questioning when the person was shown the page in the aid that answered the question [22-24]. The effects of memory aids were also evaluated in conversations and care interactions between people living with dementia and nursing aides [25-27] and spousal caregivers [28,29]. The overall conclusion of these studies was that reading is believed to be an overlearned behavior and a preserved skill and that personalized text has the potential to moderate the effects of memory loss [30]. Therefore, *My PATI* features incorporate individualized text and content to support meaningful engagement for the person living with dementia and their caregiver [31].

From the perspective of a speech-language pathologist, *MY PATI* clearly provides an approach to treatment that is rehabilitative in nature. Communication rehabilitation for people living with dementia encompasses all treatments geared toward providing the client with the skills or access to strategies that will assist them to regain or compensate for what has been disrupted [32,33]. A major objective of *MY PATI* was to compensate for communication functions to the greatest extent possible about daily preferences. This is instrumental for facilitating person-centered care (PCC) for people living with dementia. PCC in dementia care stems from the groundbreaking work of Kitwood [31], *Dementia Reconsidered the First Comes First,* which asserts that people living with dementia need to be involved in decision-making about their care whenever possible. It also posits that care for people living with dementia should emphasize their preferred needs, values, and life history. This is facilitated by knowing the person receiving care, which requires interpersonal relationship and communication between people living with dementia and their care providers [34-36].

As PCC has become the gold standard of care, an increasing number of technologies that support the autonomy of people living with dementia have emerged, including monitoring technologies for the safety of people living with dementia, robotics, therapeutic technologies, and various apps to support brain and mental health [37]. Hence, *My PATI* is part of a larger trend in IT to support this population. Unfortunately, there currently lacks a consensus on standards or guidelines for delivering PCC to inform technology development [34-37]. However, the development of *My PATI* has been informed by the definitions and conclusions reported by the American Geriatrics Society Expert Panel on PCC [36].

## An 8-Step Process for Developing My PATI

### Overview

Moving from the traditional paper format of an AAC device or memory aid for people living with dementia to an electronic version requires experts from multiple disciplines, including nursing, social work, physical therapy, speech therapy, and software engineering. Interdisciplinary development teams can sometimes encounter challenges when health and social scientists collaborate with engineers. For example, health and social scientists may not understand the capabilities or limitations of technologies, and engineering members may not understand the context of health and social problems being addressed. There are no easy answers on how to address these barriers. Still, an agreed-upon stepwise process that allows for regular opportunities for groups to talk about issues and share progress is helpful.

**Figure 2:**
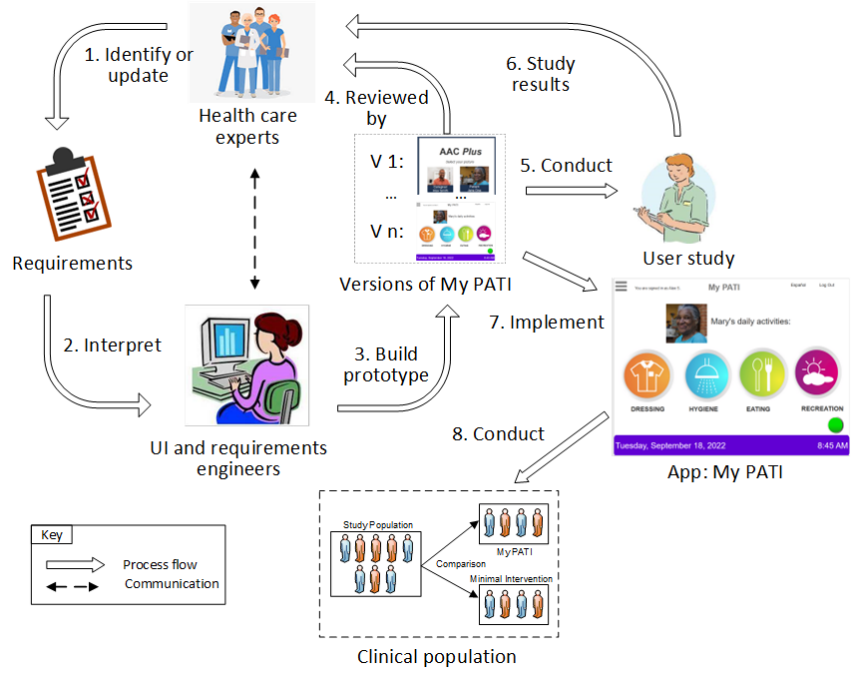
Multistep process used to develop and evaluate My Person Assisted Touchscreen Interface (My PATI).

To develop *My PATI*, our team developed and implemented an 8-step process (see Figure 2). The diagram provides a proposed communication flow for information throughout the 8 steps. The ongoing communication between the software engineers and the clinical and research team members was critical. This was a challenge as this development work was primarily done remotely during the COVID-19 pandemic. Ongoing communication occurred primarily with the use of web-based meetings, emails, and phone calls. Additionally, in-person interactions with research subjects were not permitted for a period of time due to the pandemic, which posed challenges for obtaining feedback from potential end users. Web-based focus groups and interviews were used. Beginning in the next section of this paper, we describe our multistep process that has been implemented to develop and evaluate *My PATI*.

The underlying principle for developing *My PATI* in steps 1 through 4 in Figure 2 is based on 3 software process models. These models included the incremental, throwaway prototype, and agile (a combination of incremental and prototyping, see Textbox 2 for definitions) models [38,39]. *My PATI* is a research app, thus making the requirements, by definition, inherently vague. Further adding complexity to the development process is the fact that the research team is highly interdisciplinary. The overall approach to developing the app involved first defining the software requirements using a throwaway prototype, then applying an agile approach to implement the app based on the requirements produced by the team. The throwaway prototype was critical to getting the UI correct before any implementation activities of the app starts. The agile approach (see Textbox 2) was the most suitable for the implementation stage since the health care experts and potential users (ie, customers) were frequently consulted during the implementation of *My PATI.* The approach we used for the project is supported by Dawson and Dawson [39], based on their analysis of various software process models using functionality-time graphs and combined functionality-time/cost/benefit graphs.

**Software engineering definitions.**

- An *incremental software model* produces successive increments of working, deliverable software based on partitioning of the software requirements to be implemented in each of the increments [40].
- *Software prototyping* is an activity that generally creates incomplete or minimally functional versions of a software application, usually for trying out specific new features, soliciting feedback on software requirements or user interfaces, further exploring software requirements, or gaining some other useful insight into the software [40].
- *Agile software methods* are considered lightweight methods in that they are characterized by short, iterative development cycles; frequent customer (ie, health care experts and potential users) involvement; and an emphasis on creating a demonstrable working product with each development cycle [40].

### Step 1: Identification of My PATI Requirements by Clinical Experts

The development and decisions regarding *My PATI app features* were guided primarily by the work of Bourgeois and theories on PCC, as described earlier. Early in the process, a goal of the social scientist members of the team was to provide the software engineering team with a background in PCC. This was essential as the common understanding of the challenges and strategies for promoting evidence-based PCC provides a road map and common language for our work. The engineering team was provided with articles and related videos to assist them in developing a greater understanding of the uniqueness and routine daily needs of people living with dementia. Clinical team members provided insights into functional and cognitive impairments that provided the foundation for *My PATI* features and navigation based on their experiences with patients and families. The decision to use touchscreen technology was evidence based, as touchscreens are the optimal mechanism for delivering *My PATI* to people living with dementia. The use of touchscreen technology by people living with dementia has been the focus of 2 systematic reviews [16,17], which established that people living with dementia can use personalized touchscreen technology independently and engage with touchscreen technology to improve interactions, supporting relationships and fostering PCC.

### Step 2: Requirements Interpreted by the Software Engineers

The design of the *My PATI* prototype incorporated a goal-directed and user-driven design process. In this approach, the main focus of planning and developing the system was on the goals and needs of the primary user population for each functional component or interface of the system. This resulted in providing enhanced usability and ubiquitous user experiences (UXs) for the targeted users [41]. The UI/UX experts on the software engineering team began this process by working closely with the entire team, assessing the purpose, scope, and goals for creation of the *My PATI* design. It was determined that there were 2 functional components of the system, namely (1) the capabilities of people living with dementia for communicating their preferences and feelings about their daily care activities and experiences; and (2) the capabilities of caregivers for set-up and the available customization functions of the app.

For the more detailed design, the UX experts incorporated UX design best practices, including conducting in-depth formative and substantive UX research throughout the development process [20]. For the UX research, the clinical experts of people living with dementia were the participants of the studies. This was important as it is the clinical experts who are the most knowledgeable about the primary users of the interface for people living with dementia, particularly their special cognitive and functional needs. UX research included contextual inquiries using a think-aloud protocol, clinical expert focus groups, persona and scenario creation, user case studies, and workflow analysis. The design and development studies were conducted separately with the clinical experts, caregivers, and people living with dementia. The methodologies used included participatory design using paper prototyping, wireframing and interactive prototyping, heuristic evaluation, and usability testing.

### Step 3: Engineers Built a Prototype

As described above, the software engineering team built the prototype using the requirements provided by the health care experts and interpreted by the UI/UX experts. The requirements were captured using structured natural language as use cases. The *My PATI* prototype was built using Axure [42]. Axure is a wireframing and prototyping tool that allows UI/UX designers to quickly create interactive interfaces for an app without writing a line of program code. Figure 2 shows the cycle used to develop the *My PATI* prototype, which involved steps 1, 2, 3, and 4 in the cycle. The software engineering team developed at least five versions of the prototype, with the health care and UI/UX experts reviewing each version and providing feedback to the engineering team. These versions are shown in the box labeled “Version of *My PATI*” between steps 3 and 4 and were built using the best practices in UI/UX development [20]. The first and last versions of the *My PATI* prototype are shown in Figure 2. Additional screenshots of *My PATI* are shown in Figure 1, showing how Mary and her caregiver are able to select her daily activities, such as what Mary would like for breakfast and what she would like to wear for the day.

### Step 4: Prototype Evaluated by Clinical Experts Followed by Repeating Steps 1-4

The research team then had the clinical experts review the prototype. It was initially determined that the graphic icons used for the app may not adequately reflect the feature or function that the icon represented (eg, music library and video library). The research team then consulted with an artist with UI/UX experience. The clinical team then engaged in a series of meetings with the artist to provide their clinical input about the various aspects of the icon (eg, the significance of the icon and its appropriateness for people living with dementia). The artist then redesigned the icons, which were integrated into the prototype. When this process was complete, the team continued steps 1 through 4 until consensus was reached on icon design.

### Step 5: User Study Conducted With a Person Living With Dementia and a Caregiver

User studies with both people living with dementia and caregivers have been conducted following approval from an Institutional Review Board (IRB-21-0527). For potential end user feedback, we implemented a participatory research design with persons with moderate cognitive impairment (defined as a recent Montreal Cognitive Assessment score of 10-17 or Mini-Mental State Exam score of 13-20) and caregivers at a collaborating memory clinic. The provided data revealed potential navigation issues, and our team made modifications to simplify navigation based on these findings. The protocol was structured and developed jointly by clinicians, researchers, and software engineers, and interviews were audio recorded. During the interview of the person living with dementia, we evaluated if the *My PATI* app’s features could be accessed by the participant without difficulty. Finally, we validated whether the participant’s selections were valid and reflected the true preference of the person living with dementia (as opposed to random touches on the screen). The team worked together to develop a methodology to gain insight into the validity of the person’s true preference. Our approach was to compare the selected iPad choice (in the *My PATI app*) to the actual item. See Textbox 3 for an example of how the protocol validated respondent food choice.

To date, for these participants, there was agreement between the snack choice made with the *My PATI* interface and the snack choice made when the person living with dementia was presented with the actual snack by the interviewer. There was agreement for the majority of validation tests. More data from more participants are needed and are planned. To date, 5 participants—3 caregivers and 2 people living with dementia—have been able to navigate through the app and perform tasks with ongoing guidance and support. A barrier to this usability testing has been that the information in the current *My PATI* prototype is not “personalized” (eg, generic pictures of food, clothing, and activities). A pilot is planned to test *My PATI* with personalized information (step 7).

**Instructions on the interview protocol for user study.**

Interviewer: “Now I am going to ask you about things you ordinarily like to eat and drink.”

- Show the person living with dementia the 2 snack options, pointing to each but not touching the iPad.

Interviewer: “What do you ordinarily like to eat?”

- Hold the iPad while the participant selects their choice or if the participant verbally states their response, select their response for them. Once a selection is made, put the iPad down and bring out the physical snacks (ie, identical to images on the iPad) while stating the following.

Interviewer: “I have a couple of snacks. Which of these snacks do you prefer?”

- Document the selected snack.

### Step 6: Results Interpreted by Experts and Updated

This step is similar to step 4, where the clinical experts will review the results from the user study and make recommendations for interface revision (if deemed necessary), which would be followed by steps 1-4 again.

### Step 7: My PATI Pilot Implementation

A small pilot will be conducted in preparation for the planned clinical trial (see step 8). These findings will be used to make necessary changes to *My PATI* and inform the design of a subsequent clinical trial (step 8). The team recognized from previous research [43-46] that the implementation and use of an app can present challenges. As a component of implementation, a decision was made that materials are needed to both educate the participants on technology and guide them in the use of *My PATI*; these materials will be evaluated in the pilot. Central to these implementation materials will be audiovisual presentations (available on the web) for the *My PATI* app described below. A decision was made, based on our previous research [44,46], that the technology needs to be seamlessly integrated into daily life, or it will not be viewed as helpful and be underused. It is for this reason that the “*A Day in the Life with My PATI*” implementation video is being developed. Textbox 4 provides a condensed version of the script from the video, which portrays a common daily interaction between Grandma Mary (person living with dementia) and Samantha (her granddaughter).

**Condensed script excerpt from the My Person Assisted Touchscreen Interface (My PATI) implementation video.**

Samantha: “Good Morning, Grandma. How are you feeling today?”

Grandma Mary: (smiles at Samantha)

Samantha: “Grandma, we have a doctor’s appointment later this morning. What would you like to wear today?”

- Samantha shows Mary photos of her clothes displayed on *My PATI*. Mary looks over her choices and touches the icons on the screen picturing her pink blouse and white slacks.

Samantha: “Nice choice, Grandma! You will look very pretty for the doctor. Let’s get ready.”

- Samantha helps her grandmother stand up, and they move off together to get dressed.

### Step 8: Conduct a Clinical Trial of My PATI

A trial is planned to commence in the spring of 2024 with triads of people living with dementia, caregivers, and their health care providers [47,48]. The study will investigate the impact of using *My PATI* on a number of outcomes. Two clinical sites (Miami, Florida, and Birmingham, Alabama) are collaborators for the project, and the study will enroll 60 triads at each site. The trial consists of 2 arms, where participants in both arms will continue receiving regular health care services at their respective clinics. Participants in the treatment (*My PATI*) group will receive training on how to use the device and then be asked to use the device as part of their regular daily caregiving routine for a period of 12 months. Outcome measures will be assessed at baseline, 6 months, and 12 months over the phone. Primary outcome variables include improving the quality of life for people living with dementia and their caregiver. Secondary outcome measures include depression, positive aspects of caregiving, caregiver burden, and overall health for the caregiver; depression, memory, and behavioral problems; functional linguistic communication for people living with dementia; engagement of people living with dementia and their caregivers; and provider perceptions of the intervention. All user data for *My PATI* by all participants will be tracked and analyzed*.* Full details about the outcome variable measures to be used and the research design can be accessed at ClinicalTrials.gov (NCT04571502). Results are anticipated to be available in 2025.

## Limitations

There are several limitations of the 8-step approach presented in this paper and adjustments that should be made if the process was repeated. First, most the team had previously worked together on the development and testing of another dementia caregiver app before developing *My PATI* [44]. It is suspected that clinical and engineering teams working together for the first time would need more time to progress through the early stages of the 8-stage process. Second, due to the COVID-19 pandemic, our development process included limited collaboration with people living with dementia and their caregivers. Ideally, collaboration with these stakeholders would occur from the outset and for as often as needed. It should be noted that multiple members of the research team had experience with caregiving for someone living with dementia, concurrently or previously. These experiences were often included in the team discussions.

## Conclusions

This paper provides a road map for implementing an interprofessional practice approach to developing an evidence-based app, *My PATI*, designed for older adults diagnosed with dementia and their caregivers. An interdisciplinary team of clinicians, researchers, and software engineers collaborated to develop *My PATI* to improve the quality of life of a person diagnosed with dementia by increasing their independence and participation. *My PATI*’s 8-step developmental process is described along with hypothetical scenarios demonstrating its use*.* The authors of this paper are planning to initiate the trial in the winter of 2024. This study will investigate the effectiveness of using *My PATI* in improving the quality of life of people with Alzheimer disease and related dementias and their caregivers. The results of this study are anticipated to be available in 2025.

## Abbreviations

AAC: augmentative and alternative communication
ADL: activities of daily living
IADL: instrumental activities of daily living
My PATI: My Person Assisted Technology Interface
PCC: person-centered care
UX: user experience
UI: user interface
